# Validation of Optimum ROI Size for ^123^I-FP-CIT SPECT Imaging Using a 3D Mathematical Cylinder Phantom

**DOI:** 10.22038/aojnmb.2018.10638

**Published:** 2018

**Authors:** Hideo Onishi, Takayuki Sakai, Osamu Shiromoto, Hizuru Amijima

**Affiliations:** 1 Program in Health and Welfare, Graduate School of Comprehensive Scientific Research, Prefectural University of Hiroshima, Hiroshima, Japan; 2 Department of Radiology, Kyushu Rosai Hospital, Japan Labor Health and Welfare Organization, Fukuoka, Japan; 3 Graduate School of Nursing, Hyogo University of Health Sciences, Hyougo, Japan

**Keywords:** Partial volume effect, Southampton method, Specific binding ratio

## Abstract

**Objective(s):**

The partial volume effect (PVE) of single-photon emission computed tomography (SPECT) on corpus striatum imaging is caused by the underestimation of specific binding ratio (SBR). A large ROI (region of interest) set using the Southampton method is independent of PVE for SBR. The present study aimed to determine the optimal ROI size with contrast and SBR for striatum images and validate the Southampton method using a three-dimensional mathematical cylinder (3D-MAC) phantom.

**Methods:**

We used ROIs sizes of 27, 36, 44, 51, 61, 68, and 76 mm for targets with diameters 40, 20, and 10 mm on reference and processed images reconstructed using the 3D-MAC phantom. Contrast values and SBR were compared with the theoretical values to obtain the optimal ROI size.

**Results:**

The contrast values in the ROI with diameters of 51 (target: 40 mm in diameter) and 44 (target: 20 mm in diameter) mm matched the theoretical values. However, this value did not correspond with the 10-mm-diameter target. The SBR matched the theoretical value with an ROI of > 44 mm in the 20-mm-diameter target; but, it was under- and overestimated under any other conditions.

**Conclusion:**

These results suggested that an ROI should be 2-4 folds larger than the target size without PVE, and that the Southampton method was remarkably accurate.

## Introduction

^123^I-N-ω-fluoropropyl-2β-carbomethoxy-3β-(4-iodophenyl) nortropan (^123^I-FP-CIT) single-photon emission computed tomography (SPECT) allows the visual and quantitative assessment of the striatal dopamine transporter (DAT) (1-4). The sensitivity of (^123^I-FP-CIT) SPECT for detection of nigrostriatal denaturation has been reported to be 90% (5). The detectability of this state increases when striatal accumulation decreases in proportion to Parkinsonism exacerbation (6-7). The calculation of specific binding ratio (SBR) is a significant part of this study. 

If the corpus striatum on SPECT images blurs the partial volume effect (PVE) more widely than the actual corpus striatum capacity, the accuracy would decrease, and SBR would be underestimated (8-9). Bolt et al. (10) considered that the underestimated regions of interest (ROI) are affected by the size of the ROI and suggested a novel method for setting a uniformly shaped ROI for the corpus striatum. 

They estimated SBR using the Southampton method (10). The SBR does not depend on the spatial resolution of SPECT images, and they reported improved measurement accuracy. Furthermore, since the area of the ROI exceeded the volume of the corpus striatum, they concluded that a large ROI includes background counts without a corpus striatum count.

Bolt et al. (11) reported that the SBR differs across institutions due to differences in collimators that are specific to SPECT devices (12). They also suggested that this was caused by the effects of scatter and septal penetration by I-123 gamma ray energy. However, they did not address the effects of spatial resolution that are associated with PVE. Furthermore, they introduced an analysis based on the results of Fleming et al. (13) without evidence specifying the size of the area of interest set on the corpus striatum. However, the ability of the Southampton method in reducing PVE without depending on spatial resolution has not been investigated. 

This study aimed to determine the validity of the Southampton method by investigating the amount of PVE reduction using ROIs of various sizes and a 3D-mathematical cylinder (3D-MAC) phantom (14) with a circular target of known volume. Multiple ROIs were set on a circular object (with diameters of 40, 20, and 10 mm) on the 3D-MAC phantom, and then contrast values and SBR were compared with a theoretical value to optimize the ROI size.

## Methods

***Study design***

**Figure 1:**
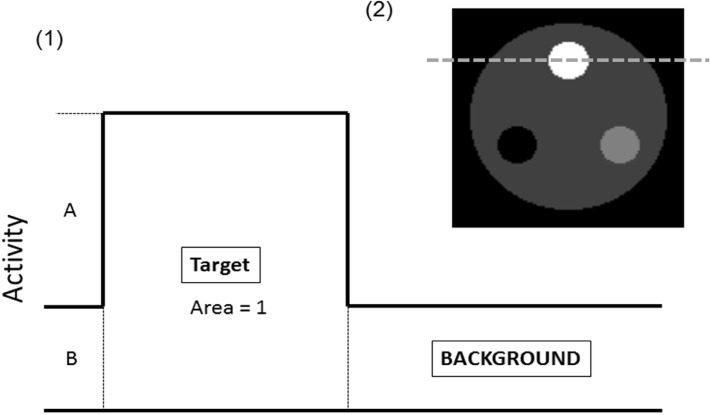
Reference image of three-dimensional mathematical cylinder (3D-MAC) phantom

We postulated that target A (Bq per unit area) exists in background (BG) B (Bq per unit area) in Figure 1. The area of the target is Ta and the area of the ROI is Ra. The area is defined by pixels in the target and ROI. The R is the value obtained by dividing the area of the ROI by the area of the target. 

(Eq. 1) R=Ra/Ta

Equation 2 describes the derivation of contrast value (CONV) as:

(Eq. 2) CONV=(C_A_-C_B_)/C_B_

where C_A _is the total count in the ROI, and C_B_ is the background count. 

**Figure 2:**
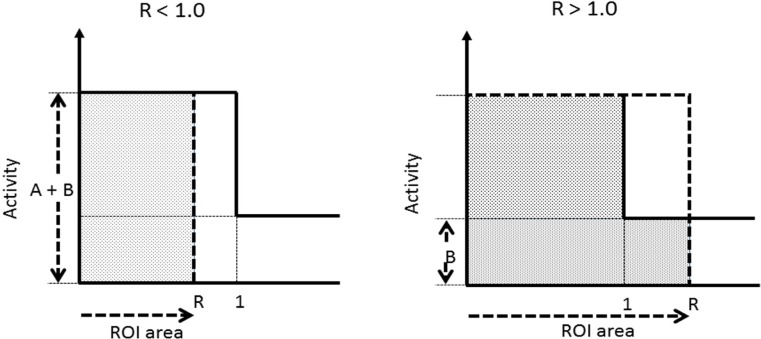
Illustration of contrast value by conjugation of target and region of interest size (R<1.0, R>1.0

Figure 2 illustrates separate conditions, namely, R ≤ 1.0 and > 1.0:

C_A_=A×R, C_B_=B×R

R ≤ 1.0: CONV=(A×R + B×R - B×R)/B×R

= A/B

R > 1.0: CONV=(A×1 + B×1 - B×1)/B×R

 = (A/B) × (1/R)

If the ROI is large, 1/R is a correction term. The SBR that is an index of the potential of corpus striatum binding in the Southampton method is defined by Equation 3 as follows:

 (Eq. 3) SBR=(1/V_s_) × {Ct_VOI_/C_r_-V_VOI_}

where Ct_VOI_ is the total count in the ROI, C_r_ is the reference count (background), V_VOI_ is the size of the ROI, and V_s_ is the size of corpus striatum (target). Therefore: 

SBR=(1/1) × {(A×1+B×R)/B-R}

=A/B 

When R ≤ 1.0, SBR (=A/B) is the same as CONV (=A/B), and when R > 1.0, R must be multiplied by CONV to determine the SBR.

***Target of 3D-MAC*** ***phantom***

We used a 3D-MAC phantom 200 mm in diameter (φ) and 200 mm in length with three embedded stacks of five 30-mm-long cylinders with diameters of 4, 10, 20, 40, and 60 mm. The relative radioactivity values were 1, 0, 2, and 4 for the background, cold stack, and two hot stacks, respectively. The 3D-MAC phantom has ideal image and SPECT projection datasets, including scatter correction, attenuation correction, and resolution recovery effects. The scintillation camera models were generated using the electron γ-shower simulation program (15-16). 

**Figure 3:**
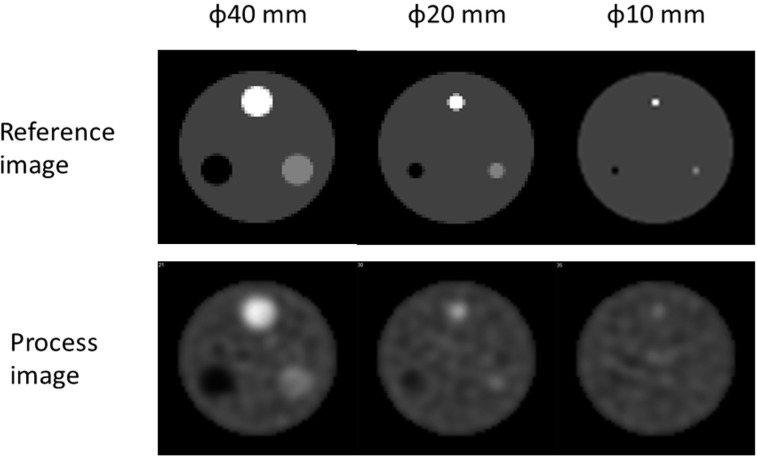
Reference and processed images.

We defined the reference image as the ideal image. The processed images were respectively reconstructed from the projection datasets (Figure 3). The reference images were obtained using a 128×128 matrix and 2 mm pixels. The projection datasets were simulated using a low-energy high-resolution collimator (128×128 matrix, 2 mm pixels; 120 projections). The radioactivity was assumed to be generated from technetium-99m (140 keV single-photon peak) using primary photons. 

***Reconstruction and ROI setting***

The images were preprocessed using a Butterworth filter (order: 8; cutoff frequency: 0.5 cycles/cm). Furthermore, they were reconstructed using ordered-subset expectation maximization (OSEM). We performed 11 iterations with 8 subsets. The attenuation was corrected using the iterative Chang method with a linear attenuation coefficient of 0.15 cm^-1^ and no scatter correction. 

**Figure 4:**
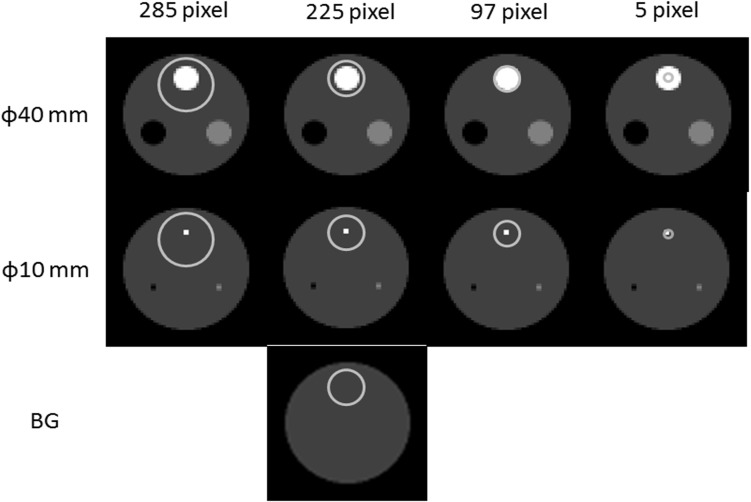
Setting regions of interest on targets.

Figure 4 shows regions-of-interest (ROI) set on the reference and processed images. The diameter and the number of pixel of the targets and ROIs are presented in Table 1. We arranged the ROI setting so that the center of the ROI coincided with the center of the target on the reference and process images. The background ROI was 225 pixels and was arranged in the same position as the target ROI on the non-target image slice. We derived with reconstruction and data processing using the prominence processor (Prominence Conference).

***Evaluation***

The reference and processed images were selected to assess the targets (SBR, 3.0) in which the relative radioactivity of the background was 4.0 and the slices had the diameters of 40, 20, and 10 mm (Figure 3). The full width at half maximums (FWHMs) were 4.0 and 20.4 mm in the reference and processed images, respectively. We calculated the measured and theoretical CONV and SBR. Furthermore, the error for SBR was evaluated in the reference and processed images.

We measured CONV and SBR using the following equation:

 (Eq. 4) CONV=([C_A_-C_BG_]/C_BG_)SBR=CONV×R

where C_A _and C_BG _are the average counts of the target and background, respectively, and R is defined as the size of the ROI divided by the size of the target. 

Because the radioactivity concentration ratio of the target and background was 4, CONV_theoretical _and SBR_theoretical_ were as follows:

CONV_theoretical_=3/R

(Eq. 5) SBR_ theoretical_=3

The SBR was 3.0 as shown in Equation 5.

## Results

***Reference images***

**Figure 5:**
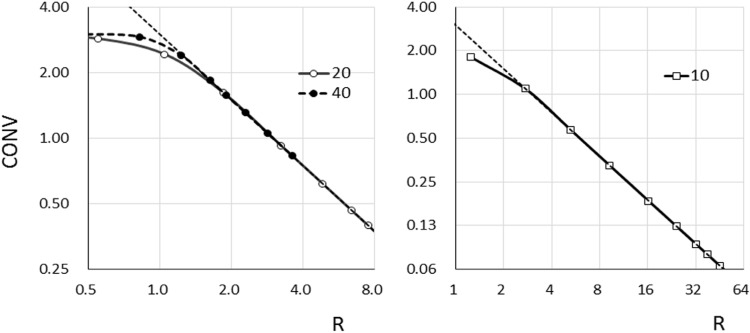
Plot of profiles through contrast value for targets with diameters of 40, 20, and 10 mm as function of R in reference images; dashed line: theoretical contrast value

Figure 5 shows the CONVs of the target with diameters of 40, 20, and 10 mm as the function of R in the reference image. The relationship between CONV and R was indicated by a power function. The CONVs were decreased with R of > 1.0 in the 40- and 20-mm-diameter targets. When R was > 1.23 (diameter of 40 mm), 1.85 (diameter of 20 mm) and 2.98 (diameter of 10 mm), the CONVs of 40-, 20-, and 10-mm-diameter targets corresponded to the theoretical values, respectively. 

***Processed images***

**Figure 6.**
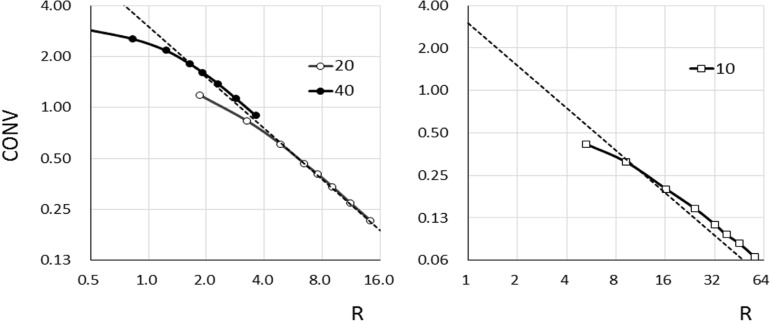
Plot of contrast value (CONV) versus R for targets with diameters of 40, 20, and 10 mm in processed images; dashed line: theoretical contrast value

**Figure 7:**
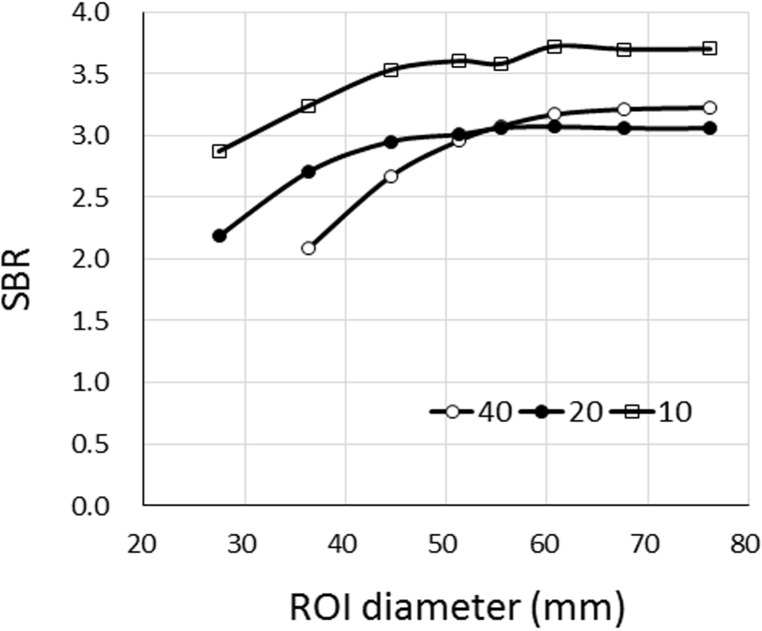
Specific binding ratio as function of region of interest (ROI) diameter in processed images of targets with diameters of 40, 20, and 10 mm; theoretical SBR is 3.0; The SBR of 10-mm-diameter target is not the true value and was overestimated when ROI diameter was > 3.5 mm

Figure 6 shows the CONVs of the target diameters of 40, 20, and 10 mm as a function of R in the processed image. Figure 6 shows theoretical values of CONV at R < 1.63 and < 4.85 for target diameters of 40- and 20-mm in processed image, respectively. Figure 7 shows the SBRs of the 40-, 20-, and 10-mm-diameter targets as a function of the diameter of the ROI. The SBR of the 40-mm-diameter target was only the theoretical SBR (=3.0) when the ROI diameter was 50 mm. With the ROI diameters of < 55 or >55 mm, underestimation and overestimation errors were both < 7.0%. 

The SBR of the 20 mm-diameter target was the theoretical value over an ROI diameter of 44 mm, and SBR was underestimated at a ROI diameter of < 44 mm. The errors of the theoretical SBR for the 40 mm and 20 mm diameters and *t* of 3.0 were < 7% and < 3%, respectively. On the other hand, R represented the theoretical value only when CONVs of the 10-mm-diameter targets were 9.25 and 16.3. The SBR error of the 10-mm-diameter target was 10%, except in the situation mentioned above. 

## Discussion

Soret et al. (8, 17) reported that the target volume is affected by PVE. We commonly considered that PVE has 40 < 20 < 10 mm-diameter targets in general. We defined the lower limit of R in congruence with the theoretical value as optimized R. As displayed in Figure 4, a lower R indicated the theoretical value of CONV in the reference images of 9.25, 4.58, and 1.63 for the targets with diameters of 10, 20, and 40 mm, respectively. The optimized R tended to increase with the reduction of the target diameter. 

The lower limits of R were 4.85 and 1.63 (diameters of 20 and 40 mm, respectively) in the processed images, and the optimized R increased with decreasing diameter similar to that in the reference images. The results suggested that the effect of PVE can be realistically described as optimized R. The spatial resolution of the image significantly impacts PVE (13). The PVE of the processed image was significantly affected because the FWHM of the processed and reference images were respectively 20.4 and 4.0 mm. 

**Table 1:** Size of targets and regions of interest

**Target**	Diameter (mm)	10	20	40								
	Pixel	4	20	79								
** ROI**	Diameter (mm)	10	15	21	27	36	44	51	55	61	68	76
	Pixel	4	11	21	37	65	97	129	151	181	225	285

(1) Profile curve of relative activity (4) on reference image in 3D-MAC phantom, (2) activity of the target is A, and background is B. Total target activity is defined as A+B

Upper and lower columns, reference and process images. Target diameters are 40, 20, and 10 mm

We set region of interest (ROI) on targets so that relative activity is 4 for background; size of ROI (5-285 pixels) and background ROI (225 pixels) set on non-target images; white circles are setting ROI

**Figure 8:**
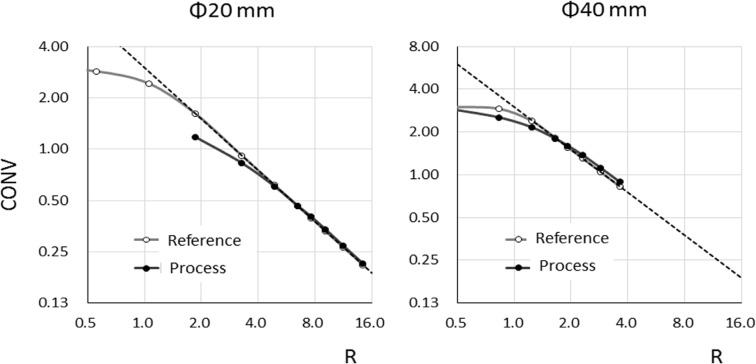
Contrast value as a function of R for reference and processed images of targets with diameters of 40 and 20 mm

**Figure 9.**
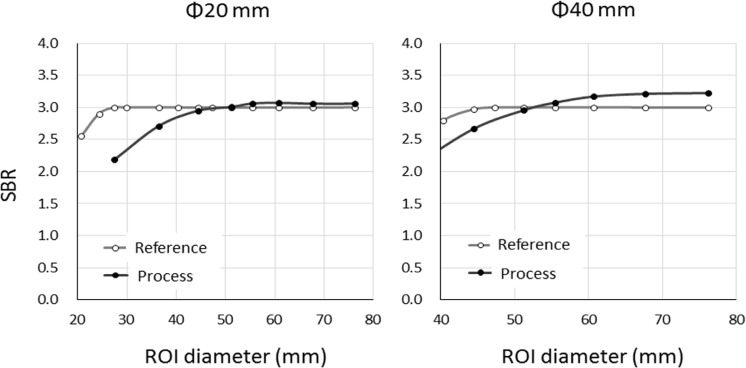
Plot of specific binding ratio (SBR) versus region of interest (ROI) diameter on reference and processed images; the SBR of targets with diameters of 20 and 40 mm in reference images show theoretical values; error for SBR was 3-7% for larger ROI

**Figure 10.**
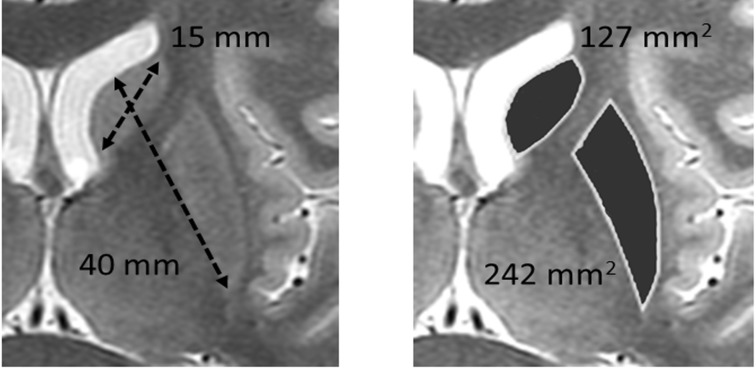
Striatal size measured on magnetic resonance images; major and minor axes are 40 and 15 mm, respectively; putamen and caudate areas are 242 and 127 mm^2^, respectively

The comparison of the relationships between CONV and R in the reference and processed images based on the target size is illustrated in Figure 8. The optimized R of the 20-mm-diameter target increased around 2.6 folds (i.e., from 1.85 to 4.85) in the reference image with degraded spatial resolution in the processed image. On the other hand, the effect of PVE was comparatively minor, and the optimized R of the 40-mm-diameter target increased 1.3 fold (i.e., from 1.23 to 1.63) in the reference image because this target was slightly less than 2 folds the size of the FWHM.

Therefore, the PVE effect occurred as the lower limit of R even if the PVE resulted from a different factor. The PVE affected the ROI size for the conjugation of optimized R in this manner, but some points were not affected by PVE, and the measurements indicated the true value if the ROI size exceeded the lower limit. The results indicated that the ROI of the same dimension could be set on images with different spatial resolutions. 

The SBR of the 10-mm-diameter target indicated an overestimation in Figure 6. There was a difference between the theoretical and actual measurement of the R value. Figure 9 shows the SBR of the targets with diameters of 40 and 20 mm as the function of the ROI diameter in the reference and processed images. The SBR obtained from the ROI on the 51-mm-diameter target was the theoretical value for the 40-mm-diameter target on the combined reference and processed images. 

Therefore, it could be said that SBR of the processed image would be identical to the reference image SBR if the size of the ROI in the processed image is appropriate. In other words, these results suggested that an appropriate ROI can facilitate the determination of an accurate quantitative value regardless of the image spatial resolution. 

We reproduced the results described above for clinical SBR. To this end, we measured a striatal area on magnetic resonance images (MRI) (Figure 10) with a major axis of 40 mm and a width of 15 mm. Due to the impossibility of the implementation of a simple comparison between a striatal and a circular subject, we showed the striatal dimension as an area on the MRI (striatal=369 mm^2^). The area of the 20-mm-diameter target was 314 mm^2^, which was similar to that of the striatum. 

Bolt et al. (10) suggested a pentagonal ROI (2809 mm^2^) as the striatum. The R is 8.95 if the pentagonal ROI is applied to the 20-mm-diameter targets. This R is higher than the optimized R, which could estimate the theoretical value of the processed image. Therefore, we consider that the size of the Southampton ROI is sufficient for the 20-mm-diameter targets.

If the Southampton ROI is applied to the whole corpus striatum, the ROI volume is 123.6 mL. The striatal volume is 11.2 mL and R is 11.0. Therefore, the ROI of the Southampton method is sufficiently larger than the striatal volume. In addition, we assumed that the Southampton ROI was effective in the study of SPECT imaging of the clinical striatum. In addition, the spatial resolution was higher for the clinical SPECT than that of the processed image (FWHM=20.4). It was considered that the Southampton ROI was effective in the clinical SPECT image of the corpus striatum. The SBR computed from the Southampton ROI was appropriate.

Soret et al. (8) ascribed PVE to two factors by stating that spatial resolution is determined by the sampling pitch of the image and the assembly of the specific devices (scintillator and collimator). We evaluated the PVE for sampling pitch using a reference image, and assessed a specific device using the processed image. The SBR of the reference image reflected the theoretical value, whereas the processed image had many errors. These results indicated that PVE cannot be removed by specific devices. It seems that this error arises due to the differences in the performance of these systems among institutions. 

**Limitations of the study**

One of the limitations of this study was the non-use of I-123 agent and the sole utilization of Tc-99m agent for the evaluation of the processed image. It was assumed that the difference of the agent could be eliminated by using only the main photo peak, which excluded the effect of scatter radiation for the processed image. Furthermore, this effect was canceled in order that SBR is calculated as the ratio.

Other limitation of this study was the absence of additive scatter data in the processed image. This excluded the effect of scatter, and we provided a reference in an effort to prove the validity of our hypothesis. However, it is necessary to assess the added effect of scatter in future as described by Soret et al. (18). It seems that evaluation through 3D iterative reconstruction techniques, including resolution recovery, depends on the generated image (19). Therefore, the voxel of interest-based analysis can be suitable for Southampton method.

## Conclusion

The RIO should be set to a size that is 2-4 folds larger than that of the target to remove the PVE from the images and systems on the SBR derived from the ^123^I-FT-CIT SPECT images. The present findings confirm the validity of the ROI obtained by the Southampton method. The findings revealed that even a large ROI did not remove the errors between the devices.
